# Proportion of kindergarten children meeting the WHO guidelines on physical activity, sedentary behaviour and sleep and associations with adiposity in urban Beijing

**DOI:** 10.1186/s12887-020-1969-6

**Published:** 2020-02-15

**Authors:** Hongyan Guan, Zhiguang Zhang, Bo Wang, Anthony D. Okely, Meiling Tong, Jianxin Wu, Ting Zhang

**Affiliations:** 1 Capital Institute of Pediatrics, 2 Yabao Rd, Beijing, 100020 China; 2 Beijing Municipal Key Laboratory of Child Development and Nutriomics, Beijing, China; 3 Early Start, Faculty of Social Sciences, University of Wollongong, Wollongong, NSW Australia; 4 Illawarra Health and Medical Research Institute, Keiraville, NSW Australia; 5 Department of Pediatrics, Nanjing Medical University, Nanjing, China

**Keywords:** Physical activity, Sedentary behaviour, Screen time, Kindergarten, Adiposity

## Abstract

**Background:**

World Health Organisation (WHO) Guidelines on Physical Activity, Sedentary Behaviour and Sleep for Children under 5 Years of Age were released in 2019. The aim of this study was to examine the proportion of Chinese kindergarten children who met each individual guideline and each combination of the guidelines and the associations with adiposity.

**Methods:**

Participants were 254 kindergarten children aged 4.1–6.3 years recruited from three kindergartens in urban area of Beijing. Physical activity, sedentary behaviour and sleep duration were assessed using 24-h accelerometry over three consecutive days. Screen time was reported by parents. Weight and height were measured; and children were categorised into normal weight and overweight/obese groups according to the WHO age- and sex- specific criteria. Frequency analyses were performed to examine the proportion of children meeting individual and combination of these guidelines. Logistics regression analyses were conducted to examine the associations between guideline compliance and adiposity.

**Results:**

The proportion of children who met the physical activity (≥ 3 h daily physical activity, including ≥1 h daily moderate- to vigorous-intensity physical activity (MVPA)), sedentary screen time (< 1 h/day), and sleep guidelines (10-13 h/day) were 65.4, 88.2 and 29.5%, respectively; only 15.0% met all three guidelines and 2.7% did not meet any of the guidelines. Not meeting the physical activity guideline, sleep guideline, or combination of any two guidelines, or all three guidelines was not associated with overweight or obesity; however, children who did not meet the sedentary screen time guidelines were at higher risk for overweight and obesity (odds ratio = 3.76, 95% CI: 1.50–9.45).

**Conclusions:**

In our study, only a small proportion of children met all three guidelines. Most Chinese kindergarten children met physical activity guidelines or screen time guidelines, whereas fewer children met sleep guideline. Not meeting sedentary screen time guidelines was associated with adiposity, which warrant further interventions for limiting screen time in young children.

## Background

Childhood overweight and obesity are significant global public health issues. Once considered a high-income country problem, the prevalence of overweight and obesity increased considerably in low- and middle-income countries, particularly in urban settings [1]. In 2016, over 41 million children under five years were overweight or obese and almost three quarters of these live in Asia and Africa [2]. China, a middle- income country, has the largest population in the world. With the rapid economic growth over past decades, there has been an increase in the number of children being overweight and obese in this country [3];and by 2030, China is estimated to have the highest number of obese children aged 5 to 19 (62 million) in the world [4]. In 2010, the prevalence of overweight in Chinese children under 5 years of age was 6.6% [5]; In 2014, this number was estimated between 5.0–9.9% in a World Health Organization (WHO) report, which was similar to the prevenance in high-income countries, such as the US and Australia [6]. These are concerning, given that overweight and obesity can profoundly affect children’s physical health, social and emotional well-being, and self-esteem [7–10]. Moreover, overweight and obesity in the early years can track into later childhood and adulthood [11, 12]. This, in turn, may lead to long-term health consequences, including cardiovascular disease, hypertension, type 2 diabetes mellitus, hyperlipidaemia, stroke, certain cancers, sleep apnea, liver and gall bladder disease, osteoarthritis, and gynaecological problems [13]. These findings reinforce the need to prevent overweight and obesity in young children, especially those living in low- and middle-income countries undergoing rapid economic transformation.

Evidence suggests movement behaviours (i.e., physical activity, sedentary behaviour and sleep) are associated with adiposity in childhood [14]. In this light, young children will benefit from more physical activity, less sedentary behaviour (especially sedentary screen time) and adequate sleep to support a healthy level of adiposity [15–17]. As physical activity, sedentary behaviour and sleep represent a movement continuum (from high to low/none) and daily time allocation of these behaviours constitute a 24-h period, there is a paradigm shift in thinking regarding these behaviours from a focus on a single behaviour to the integration of these behaviours for maximum health and development outcomes, including adiposity [18, 19]. This has been acknowledged in recently developed 24-h movement behaviour guidelines in several countries (e.g., Australia, South Africa and Canada) [20, 21] and in the newly released World Health Organization (WHO) guidelines on physical activity, sedentary behaviour and sleep for children under 5 years of age [22].

Following the release of the Australian and Canadian 24-h movement behaviours guidelines, several studies from high-income countries (e.g., Australia, Canada, Sweden, Belgium) were published, examining the proportion of young children who met the guidelines and if compliance was associated with adiposity [23–26]. With the release of the WHO global guidelines in April 2019, more research is needed in low- and middle-income countries, given that many of these countries will use the WHO global guidelines and also given the increasing prevalence of overweight and obesity in young children in these countries.

Little research has been conducted in China on movement behaviours and their associations with adiposity in kindergarten children (3–6 years), despite the high prevalence of overweight and obese in children of this age group. Therefore, the purpose of this study was to examine i) the proportion of Chinese kindergarten children meeting each individual and each combination of the recently released WHO global guidelines for physical activity (≥ 3 h daily physical activity, including ≥1 h daily moderate-to-vigorous physical activity (MVPA)), screen time (≤1 h/day) and sleep duration (10–13 h/day); and ii) the associations between guideline compliance and adiposity. In China, children attending kindergartens are generally between 3 and 6 year olds; the national sport guidelines for pre-schoolers applies to this age range, and national physical activity guideline for school-aged children and adolescents applies for the 6–17 year olds [27, 28]. Therefore, although the WHO guidelines are recommended for children under five years, in the current study, 5–6 year-old children enrolled in the kindergartens were also recruited, as children of this age group are considered pre-schoolers in China and in the kindergartens’ practice, it is recommended that the WHO guidelines would apply to them.

## Methods

### Recruitment and participants

This study was conducted in Dongcheng District of Beijing. which is a relative affluent urban area. The per capita disposable income of this district was 75,574 RMB (equal to 10,696 USD) (in 2018 (compared to 67,990 RMB (equal to 9623 USD) for Beijing and ¥39,251(equal to 5593 USD) for China in the same year) [29, 30]. A convenience sample of three kindergartens located in the district was recruited in this cross-sectional study.

Considering that 3-year-old children were newly enrolled and may experience separation anxiety, we decided exclude children of this age, to avoid further increasing their anxiety. Therefore, children were eligible to participate if they were ≥ 4.0 years-of-age and generally healthy. Children were ineligible if they had medication or medical diagnosis of physical or mental impairment (except for overweight or obesity). Prior to participation in the study, all eligible children’s parents/guardians were provided with paper versions of information sheets and consent forms. Data collection was conducted between October to December 2018.

This study was approved by the Capital Institute of Pediatrics’ Human Research Ethics Committee (No.SHERLL2018001). Informed written consent was obtained from children’s parents or guardians.

### Measures

#### Physical activity, sedentary behaviour and sleep

Physical activity, sedentary time and sleep were objectively measured using waist-worn accelerometers (Actigraph GT9X). The Actigraph accelerometer has established validity and reliability in preschool-aged children [31]. Participants were asked to wear the monitors, attached on the right hip, for 24-h over three consecutive days, except for water-based activities. In addition, during this period, parents and educators were asked to complete an activity log, in which nap(s), bedtime, wake-up time and accelerometer non-wear time were recorded for each child. Accelerometer data were collected using a sampling rate of 30 Hz and re-integrated into 15-s epochs for analyses [32]. Participants had to have at least one 24-h period of accelerometer data to be included in the analyses [26].

In line with previous studies [26, 33], accelerometry data were visually inspected minute by minute, in consultation with the activity logs, to identify nap and nighttime sleep duration. Specifically, the nap onset/bedtime was initially located when a switch in the accelerometer output from the inclinometer sitting or standing to inclinometer lying or off was detected, which approximately corresponded with the nap onset registered in the corresponding activity log. The onset was then identified as the first minute followed by at least 10 consecutive minutes with a vector magnitude of zero in the output. The nap offset/wake-up time was firstly located when a switch in the output from inclinometer lying or off to inclinometer sitting or standing was detected, which corresponded with the nap offset registered in the activity log. The offset was then identified as the first minute followed by at least 10 consecutive minutes with a vector magnitude > 0 in the output. Nap duration was calculated as the period between the nap onset time and the offset time. Similarly, nighttime sleep duration was calculated as the period between bedtime and wake-time. Total sleep duration was calculated as the sum of the nighttime sleep duration and nap(s) duration. For the calculation of sleep duration, wake after sleep onset was not included.

After identification of nap(s) and nighttime sleep duration, these periods were removed from the accelerometer data, which were then analysed by an automated data reduction program (ActiLife Software, Version 6.13.3 for Windows). Non-wear awake time was flagged as 20 min of consecutive zero counts [32] and excluded from the analysis. Accelerometer data were reduced using age-appropriated cut-point to calculate sedentary behaviour (≤25 counts/15 s), low light-intensity physical activity (26–199 counts/15 s), high light-intensity physical activity (200–419 counts/15 s) and MVPA (≥420 counts/15 s) [31, 34]. Since activity levels between 25 and 199 counts/ 15 s are low-intensity activity, such as standing, including these activity levels may lead to an overestimate of physical activity guideline compliance [35]. Therefore, total physical activity was calculated as: high light-intensity physical activity + MVPA, following previous studies [34, 35].

#### Screen time

Screen time was assess using the parent-report questionnaire, with the question: How long was your child’s screen time (e.g., watching television, using iPad or phone, etc) on a typical weekday, a typical Saturday and a typical Sunday, respectively? Subsequently, the child’s average daily screen time was calculated (average daily screen time = (weekday screen time *5 + Saturday screen time + Sunday screen time)/7).

#### Operational definitions of the WHO global guidelines

##### Physical activity

Participants were categorized as meeting the physical activity guideline if they had an average of at least 3 h of total physical activity (including high light-intensity physical activity and MVPA) per day, of which at least 1 h was MVPA.

##### Screen time

Participants were categorized as meeting the screen time guideline if they had an average of no more than 1 h of screen time per day.

##### Sleep

Participants were categorized as meeting the sleep guideline if their average sleep duration was between 10 to 13 h per day.

#### Adiposity

Body weight and height were measured according to standard procedures [36]. Body height was measured to the nearest 0.1 cm in bare or stocking feet with the child standing upright against a portable stadiometer (Seca 217 Hamburg, Germany). Body weight was measured to the nearest 0.10 kg, lightly dressed using a portable electronic weight scale (Seca 899 Hamburg, Germany). Body mass index (BMI) was then calculated as weight(kg)/height(m^2^). BMI z-score were calculated according to the WHO age- and sex- specific criteria [37, 38]. For participants 5 years and below, overweight and obesity were classified as BMI z-score above 2 standard deviation and above 3 standard deviation, respectively; For participant over five years, overweight and obesity were classified as BMI z-score above 1 standard deviation and above 2 standard deviation, respectively [39]. Participants were then categorised as two groups: normal weight group or overweight/obese group.

### Covariates

#### Child age and sex

Child age and sex were assessed by parental questionnaires.

### Statistical analyses

Descriptive statistics, including means and standard deviations or percentages, were calculated for participants’ demographic characteristics, adiposity, average daily time spent on movement behaviours (accelerometer-derived data), average daily screen time and consistent sitting for no more than one hour at a time. Frequency analyses were performed to examine the proportion of kindergarten children meeting physical activity guideline, screen time guideline and sleep guideline or the combination of these guidelines. The differences in descriptive characteristics between the analytic sample and children who were excluded from the analyses due to missing data of interest were performed using student t-test. Logistics regression analyses were then conducted to examine the associations between not meeting (versus meeting) single or combination of each guidelines and odds ratios for being overweight and obese in the sample, before and after adjusted for children’s age and sex. Considering the applicable age range for the WHO guidelines, sensitivity analyses were further conducted in children under five years using the above models.

Statistical significance was set at *P* value < 0.05. Data analyses was performed using IBM SPSS, version 24.0 (SPSS Inc., Chicago, IL, USA).

## Results

The parents/guardians of 393 children agreed to participate the study; of these, 63 dropped out due to personal reasons (e.g., felt uncomfortable to wear the accelerometer or sick) before data were collected. Trained data collectors completed assessments (anthropometrics and accelerometer data) with 330 children and the guardians of these children were asked to completed questionnaires on children’s demographic information and screen time. 299 children had valid accelerometer data (i.e., have at least one 24-h period of wear time). Of these, 45 children did not have screen time data and were excluded from analyses. Children who were excluded did not significantly differently differ in their descriptive characteristics (i.e., age and percentage of boys), adiposity (BMI and percentage of overweight or obese children) and movement behaviours (i.e., total physical activity, MVPA, sedentary time and sleep duration) (Table S1). Therefore, a final sample of 254 children were included in the analyses.

**Fig. 1:**
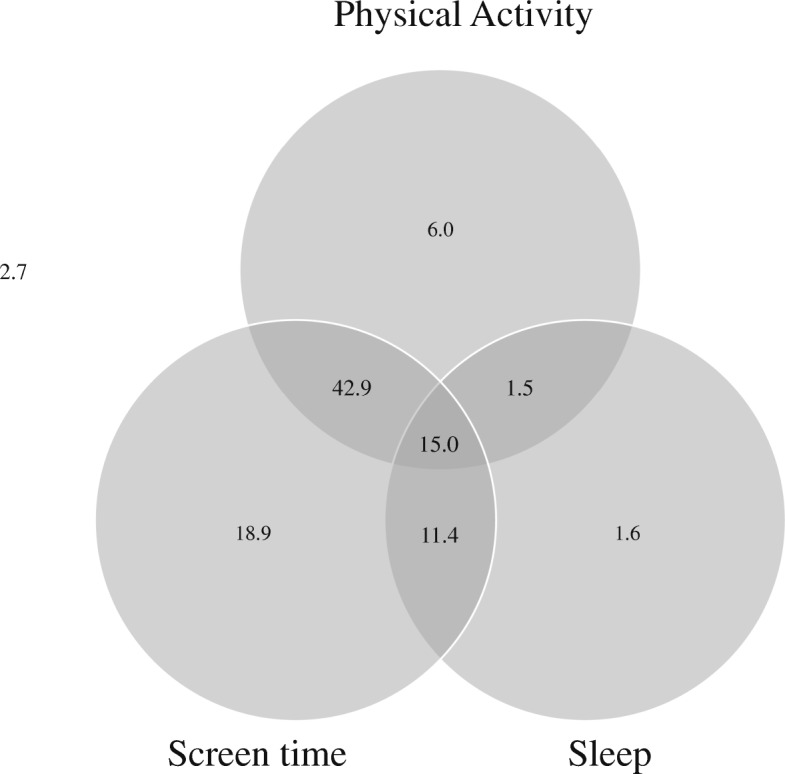
Legend: The numbers within each circle are added to the proportion of children meeting each individual guideline (i.e., 65.4% for physical activity, 88.2% for screen time and 29.5% for sleep). The total non-overlap area of each circle represents the proportion of children meeting one of the three guidelines (i.e., 6.0%+18.9%+1.6%=26.5%). The total overlap areas of two circles represents the proportion of children meeting two guidelines of the three guidelines (i.e., 42.9%+1.5%+11.4% = 55.8%). The overlap area of three circles represents the proportion of children meeting all three guidelines (i.e., 15.0%). The outside area of the circle represents the proportion of children not meeting any of the guidelines (i.e. 2.7%)

Descriptive data are presented in Table 1. Of the 254 children, 5.5% had one day (i.e., one 24-h period) of accelerometer data, 14.2% had 2 days and 80.3% had 3 or more days of accelerometer data. The proportions of toddlers meeting no guidelines, individual movement behaviour guidelines and combinations of the guidelines are shown in a Venn diagram (Fig. 1), which is an adaption from previous studies [24, 26]. The numbers within each circle added to the proportion of children meet individual guidelines: 65.4% of children met the physical activity guideline and 88.2% met the screen time guideline, whereas only 29.5% of the children met the sleep guideline. Only 2.7% of the children did not meet any of the guidelines, 26.5% (6.0% + 18.9% + 1.6%) met one of the three guidelines, 55.8% (42.9% + 1.5% + 11.4%) met two of the three guidelines, and 15.0% met all three guidelines.

**Table 1:** Participants’ characteristics

Full sample (*n* = 254)	
Age (years), mean ± SD	5.11 ± 0.58
Sex (percentage of boys)	53.1%
BMI(kg/m^2^), mean ± SD	15.73 ± 2.04
Weight status (percentage of overweight or obese children)	16.1%
Total physical activity (hour/day), mean ± SD	3.29 ± 0.72
MVPA (hour/day), mean ± SD	1.64 ± 0.46
Low light-intensity physical activity (hour/day), mean ± SD	3.75 ± 0.54
Sedentary time (hour/day), mean ± SD	7.11 ± 1.02
Screen time (hour/day), mean ± SD	0.63 ± 0.41
Total sleep time (hour/day), mean ± SD	9.66 ± 0.61
Accelerometer wear time (hour/day), mean ± SD	21.53 ± 1.12

Abbreviation: *SD* standard deviation, *BMI* body mass index, *MVPA* moderate-to-vigorous physical activity

Table 2 shows the associations between not meeting (versus meeting) single or combinations of guidelines and adiposity in the sample. Compared to children who met the screen time guideline, those who did not meet the guideline were more likely to be overweight or obese (odds ratio = 3.76, 95% CI: 1.50–9.45). Not meeting the physical activity guideline, sleep guideline, the combination of any two guidelines, or all three guidelines was not associated with overweight or obesity in the sample.

**Table 2:** The associations between not meeting (vs. meeting) single or combination of each guidelines and odds ratios for being overweight and obese (*n* = 254)

	Unadjusted models		Adjusted models	
	OR (95%CI)	*P* value	OR (95%CI)	*P* value
Not meeting (vs. meeting) the following guidelines:				
PA	0.56 (0.26,1.20)	0.136	0.68 (0.31,1.52)	0.351
Screen	**3.11 (1.33,7.27)**	0.009*	**3.76 (1.50,9.45)**	0.005*
Sleep	1.36 (0.63,2.94)	0.432	0.95 (0.42,2.16)	0.905
PA+ Screen	0.93 (0.49,1.91)	0.968	1.19 (0.58,2.43)	0.642
PA+ Sleep	0.78 (0.33,1.84)	0.576	0.56 (0.23,1.41)	0.220
Screen + Sleep	1.33 (0.60,2.96)	0.483	0.96 (0.41,2.24)	0.928
All three guidelines	0.83 (0.34,2.03)	0.679	0.61 (0.23,1.58)	0.306

Abbreviation: *OR* odds ratio, *CI* confidence interval, *PA* physical activity

Note: bold font indicate significance

In the adjusted models, age and sex were adjusted

*P < 0.05

Sensitivity analyses in children under five years shows that children under 5 years had slightly lower MVPA but slightly higher low light-intensity physical activity and sedentary time; the proportion of children being overweight and obese was also lower than those aged 5 years and above (Table S2). However, guideline compliance (Table S3) and the associations with adiposity (Table S4) in both age group.

## Discussion

The present study, to the best of our knowledge, is the first study examining the proportion of Chinese children meeting the newly developed WHO Global guidelines on physical activity, screen time and sleep for children under the age of five years, as well as the associations between guideline compliance and adiposity, using objective measures (i.e., accelerometry) of the movement behaviours. In our study, more than half of the kindergarten children met physical activity guidelines (65.4%) or screen time guidelines (88.2%), while less than one third met sleep guideline (29.5%); Only 15.0% met all three guidelines. Kindergarten children who did not meeting screen time guideline were at higher risk for overweight and obesity; however, not meeting physical activity guideline, sleep guideline, the combination of specific two guidelines, or all three guidelines was not associated with overweight or obesity.

The proportion of children meeting the physical activity guidelines varies across countries. Our finding were close to one Canadian study with 803 pre-schoolers (61.8%) [24], whereases the proportion of pre-schoolers meeting physical activity guideline were much lower (19.3 and 11.0% respectively) in another Canadian (*n* = 539) [40] and Belgian study (*n* = 595) [25] but higher (93.1%) in one Australian study (*n* = 248) [41]. In addition, in a recent Chinese study with 3030 pre-schoolers, 72.9% of the children had at least 1 h of MVPA and 35.3% had at least 3 h of total physical activity [42]. The variety in the proportion across studies may be primarily due to the differences in accelerometer data collection (e.g., different type of physical activity monitors (e.g., Actical vs. Actigraph), hip vs. wrist wearing, data collected in 15-s epoch vs. in 60 s epoch) and data reduction (e.g., using different cut-point for light physical activity/MVPA) between studies, which would result in different estimates of physical activity [40]. For example, the mentioned Australian study used similar procedures (i.e., 24-h accelerometry over a week, waist-worn Actigraph accelerometers, data collected in 15 s epoch, same cut-point for MVPA) as our study but used different cut-point for light-intensity physical activity (25/15 s vs. 200 c/15 s in our study) [41]. This may explain that the similar estimates of MVPA (1.6 h vs. 1.7 h) but less total physical activity (3.3 h versus 6.2 h) in our participants and therefore a lower proportion of children meeting the physical activity guideline, compared to the Australian pre-schoolers.

In our study, the proportion of children meeting the screen time guideline was 88.2%, which is higher than the proportion of pre-schoolers in high-income countries (ranging from 17.3–63.0%) [23–25, 40, 41]. We found that kindergarten children not meeting the screen time guideline were three times more likely to be overweight or obesity than those meeting the guideline. This is supported by evidence in children and adolescences, which suggests that excessive screen media exposure is associated with increased eating while viewing screens as well as reduced sleep duration; and these, in turn, could lead to higher adiposity [43]. However, our result did not align with findings in high-income countries, which suggest no associations between screen guidelines compliance and adiposity in preschool-aged children and toddlers [23, 24, 26, 44]. One reason for this could be the ethical or cultural difference. Also, a recent systematic review examining associations between screen time and adiposity found that longitudinal studies predominately reported unfavourable associations whereas the associations were mainly null in cross-sectional studies [16]. This suggests that the deleterious effects of screen time may take prolonged periods to manifest. Therefore, it is plausible to observe the unfavourable association in older children, where prolonged screen time habits exert a stronger influence over time. Given that the average age of our children (mean age = 5.16 ± 0.58) is slightly older than participants in most mentioned studies conducted in the high-income countries, it may, to some extent, explain the inconsistent findings. Nevertheless, our results indicate that interventions for reducing screen time are needed for kindergarten children.

Previous studies conducted in high-income countries reported that most pre-schoolers met the sleep guideline (compliance proportion ranging from 83.1–94.0%), [23–25, 40, 41] In our sample, the proportion was only 29.5%. Compared to the mentioned studies (ranging from 10.5–11.0 h), kindergarten children in our study had shorter average sleep duration (9.7 h). One possible reason for this is the older average age of our sample (5.11 years vs. 3.0–4.2 years in those studies), given that sleep decreases with age. However, this seems unlikely to be the main reason as there was no significant difference in total sleep duration or sleep guideline compliance between children aged 4.1–4.9 years (9.71 ± 0.60 h, 34.5%) and those aged 5 years and above (9.62 ± 0.61 h, 25.2%). Furthermore, total sleep duration in our sample is also lower than that reported in other Chinese studies with children of similar age [45, 46]. For example, in a study with representative sample of Chinese urban kindergarten children, during 1999–2005, the total sleep duration in 839 children aged 48–59 months and in 847 children aged 60–71 months were 11.6 h and 11.3 h respectively [45]. It should be noted that total sleep duration in these Chinese studies were assessed using subjective measures (i.e. parental questionnaire), which may be less accurate than accelerometry used in our study [17] and this may partially explain the differences. Moreover, according to a more recent study with children from nine Chinese province, it appears that there was a decline in total sleep duration in Chinese young children from 2004 to 2011, and in 2011, 75% of children aged 3–5 years slept less than 11 h per day [47]. This finding agrees with our results, indicating that nowadays Chinese young children may be at risk for short sleep duration. Nevertheless, because our sample is not nationally representative, more studies are needed to examine the sleep guideline compliance in Chinese kindergarten children, before any conclusion can be drawn.

In our sample, only a small proportion of children (15.0%) met all three movement behaviour guidelines. This is consistent with the data of preschool-age children (ranging from 5.0–18.4%) reported from previous studies conducted in high-income countries, which examined similar movement behaviour guidelines compliance [23–25, 40, 41].

Consistent with findings in young children in the high-income countries [23, 24, 26, 44], we did not find associations between adiposity and not meeting the physical activity or sleep guideline or specific combinations of two guidelines or all three guidelines. However, caution should be taken in interpreting these findings as evidence of no associations between meeting movement behaviour guidelines and adiposity. Rather, the negative effects may take relatively longer to manifest and it may not be easy to observe in young children, especially in cross-sectional studies. This is supported by the fact that studies in school-age children and adolescents predominately showed significant associations between movement behaviours and adiposity [48–51] whereas evidence in early childhood is less consistent [15–17, 52]. Furthermore, a recent international study examining the associations between meeting 24-h movement behaviour guidelines and adiposity suggests that meeting single or combined guidelines were associated with adiposity in 9–11 years children across 12 countries [53]. Longitudinal and experimental studies with longer follow-up periods are warranted to examine the associations between meeting movement behaviour guidelines and adiposity in young children.

One strength of the present study is the use of 24-h accelerometry to objectively assess movement behaviour (except for screen time), which allows us to measure prevalence as accurately as possible. However, there are several limitations in the study. First, our study is limited for the cross-sectional design which precludes establishing causality. Second, some potential confounders, such as dietary intake, that may explain adiposity in children, was not considered in the analyses. Third, 20% of children in our sample had less than 3 days of accelerometer data (but at least 1 day with 24-h wear time), which may not represent a child’s usual movement behaviour. Fourth, due to the difference in enrolment age between kindergartens in China (3–6 years) and pre-schools in some other countries (3.0–4.9 years), comparison of findings should be with cautions. Lastly, the current study was conducted in a relative affluent area of China and our sample is not nationally representative; therefore, our results may not generalize to the Chinese population of kindergarteners.

## Conclusion

In our study, only a small proportion of children (15.0%) met all three guidelines. Most Chinese kindergarten children met physical activity guidelines (65.4%) or screen time guidelines (88.2%), whereas fewer children met sleep guideline (29.5%). We did not find the associations between adiposity and the compliance of most single/combinations of guidelines. However, children who did not meeting screen time guidelines were more likely to be overweight and obese, which warrant further interventions for promoting this guideline compliance, especially for limiting screen time.

## Supplementary information

**Additional file 1: Table S1.** Comparison of characteristics between the included children and excluded children who did not have screen time data.

**Additional file 2: Table S2.** Sensitivity analyses of the descriptive data.

**Additional file 3: Table S3.** Sensitivity analyses of the guideline compliance.

**Additional file 4: Table S4.** Sensitivity analysis of the associations between not meeting (vs. meeting) single or combination of each guidelines and odds ratios for being overweight and obesity in children under 5 years.

## Data Availability

The datasets used and/or analysed during the current study are available from the corresponding author on reasonable request.

## Abbreviations

BMI: Body Mass Index
MVPA: Moderate-to-Vigorous Physical Activity
WHO: World Health Organisation
