# Supplementary material for: Proportion of kindergarten children meeting the WHO guidelines on physical activity, sedentary behaviour and sleep and associations with adiposity in urban Beijing
Source: BMC Pediatr. 2020 Feb 15;20:70. doi: 10.1186/s12887-020-1969-6 (PMC7023817; doi:10.1186/s12887-020-1969-6)
Supplement: Supplementary file 1 — Additional file 1: Table S1. Comparison of characteristics between the included children and excluded children who did not have screen time data. [file 12887_2020_1969_MOESM1_ESM.docx]

**Table S1. Comparison of characteristics between the included children and excluded children who did not have screen time data.**

| Characteristics | Included children (n=254) | Excluded children who did not have screen time data (n=45) | P value |
| --- | --- | --- | --- |
| Age (years), mean±SD | 5.12±0.58 | 5.08±0.54 | 0.662 |
| Sex (percentage of boys) | 53.1% | 53.3% | 0.982 |
| BMI(kg/m^2^) , mean±SD | 15.73±2.04 | 16.07±2.20 | 0.307 |
| Weight status (percentage of overweight or obese children) | 16.1% | 20.0% | 0.523 |
| Total physical activity (hour/day) , mean±SD | 3.29±0.72 | 3.19±0.64 | 0.373 |
| MVPA (hour/day) , mean±SD | 1.64±0.46 | 1.54±0.41 | 0.166 |
| Low light-intensity physical activity (hour/day), mean±SD | 3.75±0.54 | 3.76±0.46 | 0.916 |
| Sedentary time (hour/day) , mean±SD | 7.11±1.02 | 7.37±0.79 | 0.110 |
| Total sleep time (hour/day) , mean±SD | 9.66±0.61 | 9.52±0.61 | 0.135 |
| Accelerometer wear time (hour/day) , mean±SD | 21.53±1.12 | 21.21±1.34 | 0.089 |

Abbreviation: SD, standard deviation; BMI, body mass index; MVPA, moderate-to-vigorous physical activity

Note: Student t-test was used to examine the differences in age, BMI, total physical activity, MVPA, sedentary time, total sleep time and accelerometer wear time between included children and excluded children who did not have screen time data. Chi-square analyses was used to examine the difference in the percentage of boys and the percentage of overweight or obese children between included children sample and excluded children who did not have screen time data.
