# Supplementary material for: Proportion of kindergarten children meeting the WHO guidelines on physical activity, sedentary behaviour and sleep and associations with adiposity in urban Beijing
Source: BMC Pediatr. 2020 Feb 15;20:70. doi: 10.1186/s12887-020-1969-6 (PMC7023817; doi:10.1186/s12887-020-1969-6)
Supplement: Supplementary file 2 — Additional file 2: Table S2. Sensitivity analyses of the descriptive data. [file 12887_2020_1969_MOESM2_ESM.docx]

Table S2 Sensitivity analyses of the descriptive data

|  | Children under 5 years (n=119) | Children aged 5 years and above (n=135) | P value |
| --- | --- | --- | --- |
| Sex (percentage of boys) | 57.1% | 49.6% | 0.231 |
| BMI(kg/m^2^) , mean±SD | 15.59±1.69 | 15.86±2.30 | 0.302 |
| Weight status (percentage of overweight or obese children) | 6.7% | 24.4% | <0.001* |
| Total physical activity (hour/day) , mean±SD | 3.24±0.76 | 3.33±0.69 | 0.350 |
| MVPA (hour/day) , mean±SD | 1.55±0.48 | 1.72±0.44 | 0.005* |
| Low light-intensity physical activity (hour/day), mean±SD | 3.85±0.50 | 3.66±0.56 | 0.005* |
| Sedentary time (hour/day) , mean±SD | 6.89±0.96 | 7.30±1.04 | 0.001* |
| Screen time (hour/day) , mean±SD | 0.62±0.37 | 0.64±0.45 | 0.706 |
| Total sleep time (hour/day) , mean±SD | 9.71±0.60 | 9.63±0.61 | 0.295 |
| Accelerometer wear time (hour/day) , mean±SD | 21.65±1.16 | 21.43±1.08 | 0.124 |

Abbreviation: SD, standard deviation; BMI, body mass index; MVPA, moderate-to-vigorous physical activity

* P<0.05
