# Supplementary material for: Proportion of kindergarten children meeting the WHO guidelines on physical activity, sedentary behaviour and sleep and associations with adiposity in urban Beijing
Source: BMC Pediatr. 2020 Feb 15;20:70. doi: 10.1186/s12887-020-1969-6 (PMC7023817; doi:10.1186/s12887-020-1969-6)
Supplement: Supplementary file 3 — Additional file 3: Table S3. Sensitivity analyses of the guideline compliance. [file 12887_2020_1969_MOESM3_ESM.docx]

**Table S3 Sensitivity analyses of the guideline compliance**

| Guidelines | Children under 5 years (n=119) | Children aged 5 years and above (n=135) | P value |
| --- | --- | --- | --- |
| PA | 63.9% | 66.7% | 0.640 |
| Screen | 88.2% | 88.1% | 0.983 |
| Sleep | 34.5% | 25.2% | 0.106 |
| PA+ Screen | 57.1% | 58.5% | 0.825 |
| PA+ Sleep | 21.0% | 12.6% | 0.072 |
| Screen + Sleep | 29.4% | 26.4% | 0.303 |
| All three guidelines | 19.3% | 11.1% | 0.067 |
