# Supplementary material for: Proportion of kindergarten children meeting the WHO guidelines on physical activity, sedentary behaviour and sleep and associations with adiposity in urban Beijing
Source: BMC Pediatr. 2020 Feb 15;20:70. doi: 10.1186/s12887-020-1969-6 (PMC7023817; doi:10.1186/s12887-020-1969-6)
Supplement: Supplementary file 4 — Additional file 4: Table S4. Sensitivity analysis of the associations between not meeting (vs. meeting) single or combination of each guidelines and odds ratios for being overweight and obesity in children under 5 years. [file 12887_2020_1969_MOESM4_ESM.docx]

**Table S4. The associations between not meeting (vs. meeting) single or combination of each guidelines and odds ratios for being overweight and obesity in children under 5 years (n=119)**

|  | Unadjusted models | | Adjusted models | |
| --- | --- | --- | --- | --- |
|  | OR (95%CI) | P value | OR (95%CI) | P value |
| Not meeting (vs. meeting) the following guidelines: | | | | |
| PA | 0.24 (0.03,1.98) | 0.182 | 0.27 (0.03,2.33) | 0.233 |
| Screen | **5.46 (1.15,25.98)** | **0.033*** | **6.65 (1.25,35.45)** | **0.026*** |
| Sleep | 0.67 (0.20,3.83) | 0.851 | 0.78 (0.17,3.56) | 0.749 |
| PA+ Screen | 1.36 (0.32,5.73) | 0.674 | 1.61 (0.37,7.01) | 0.527 |
| PA+ Sleep | 0.41 (0.09,1.86) | 0.248 | 0.38 (0.08,1.82) | 0.227 |
| Screen + Sleep | 0.68 (0.15,2.99) | 0.605 | 0.59 (0.13,2.75) | 0.503 |
| All three guidelines | 0.37 (0.08,1.66) | 0.193 | 0.34 (0.07,1.63) | 0.175 |

Abbreviation: OR, odds ratio; CI, confidence interval; PA, physical activity.

Note: bold font indicate significance.

In the adjusted models, age and sex were adjusted.

*P<0.05
